# Correction to *Lancet Healthy Longev* 2022; 3: e650

**DOI:** 10.1016/S2666-7568(22)00250-1

**Published:** 2022-11

*Shawar YR, Mikton CR, Beaulieu M, Yon Y, Campo-Tena L. Clinical resource allocation for the mitigation of elder abuse. Authors’ reply.* Lancet Healthy Longev *2022; **3:** e650*—This Correspondence should have been published under a CC BY 3.0 IGO license. This correction has been made as of Nov 7, 2022.

